# Physical Activity Levels and Screen Time among Youth with Overweight/Obesity Using Mental Health Services

**DOI:** 10.3390/ijerph19042261

**Published:** 2022-02-17

**Authors:** Gerald J. Jerome, Tyler Fink, Tammy Brady, Deborah R. Young, Faith B. Dickerson, Stacy Goldsholl, Robert L. Findling, Ekaterina A. Stepanova, Ann Scheimann, Arlene T. Dalcin, Alison Terry, Joseph Gennusa, Courtney Cook, Gail L. Daumit, Nae-Yuh Wang

**Affiliations:** 1 Department of Kinesiology, Towson University, Towson, MD 21252, USA; 2 Division of General Internal Medicine, Johns Hopkins School of Medicine, Baltimore, MD 21205, USA; tfink1@jhmi.edu (T.F.); sgoldsh1@jhmi.edu (S.G.); adalcin1@jh.edu (A.T.D.); aterry11@jhmi.edu (A.T.); jgennus1@jhmi.edu (J.G.); ccook17@jhmi.edu (C.C.); gdaumit@jhmi.edu (G.L.D.); naeyuh@jhmi.edu (N.-Y.W.); 3 Department of Pediatrics, Johns Hopkins School of Medicine, Baltimore, MD 21287, USA; tbrady8@jhmi.edu (T.B.); ascheima@jhmi.edu (A.S.); 4 Kaiser Permanente Southern California, Pasadena, CA 91101, USA; deborah.r.young@kp.org; 5 Sheppard Pratt, Baltimore, MD 21204, USA; faithbdickerson@gmail.com; 6 Department of Psychiatry, Virginia Commonwealth University, Richmond, VA 23284, USA; robert.findling@vcuhealth.org (R.L.F.); ekaterina.stepanova@vcuhealth.org (E.A.S.); 7 Department of Biostatistics and Epidemiology, Johns Hopkins Bloomberg School of Public Health, Baltimore, MD 21205, USA

**Keywords:** physical activity, children, adolescent, obesity, mental health

## Abstract

Youth with mental illness have higher levels of obesity than children in the general population. Both regular physical activity and limited screen time have been recommended to reduce and prevent childhood obesity. This study examines accelerometer-based moderate–vigorous physical activity (MVPA) and screen time among youth with overweight/obesity issues who are receiving mental health care. This study looked at a 12-month weight management randomized clinical trial for overweight/obese youth aged 8–18 years who are receiving mental health services. At baseline, MVPA was assessed using accelerometers, and screen time was self-reported. Among 100 youth, 43% were female, 44% were Black, and 48% were <13 years old. In an adjusted general linear model, higher levels of MVPA were associated with the younger age group (*p* = 0.012), male participants (*p* = 0.013), and lower BMI z-scores (*p* = 0.014). In a separate model, higher screen time was associated with participants who were Black (*p* = 0.007). Achieving optimal cardiovascular health at the population level requires an understanding of the groups that are most in need of additional assistance. These data reinforce that targeted lifestyle approaches to promote increased physical activity and decreased screen time among overweight/obese youth using mental health services may need additional tailoring for sex, age, and race subgroups.

## 1. Introduction

Childhood obesity is a significant public health concern [1,2,3]. The prevalence of overweight and obesity among youth with mental illness is substantially greater than the overall pediatric prevalence [4,5,6]. The impact of childhood obesity on this group is particularly concerning for two reasons. First, there is an increased risk of adult obesity for youth with obesity [6,7]. Second, adults with serious mental illness (SMI) have high rates of obesity and related premature mortality due to cardiovascular disease (CVD), and youth with mental illness are more likely to have mental illness as an adult [8,9,10].

Physical activity has been identified as a key health behavior to address childhood obesity [11,12]. Limiting screen time has also been recommended to reduce and prevent childhood obesity [11,12]. Guidelines recommend youth accumulate 60 min/day of physical activity and limit screen time [11,12,13]. Despite the documented benefits and corresponding guidelines, the physical activity levels of youth in the United States remain low. One report estimated that only 26% of youth engage in 60 min/day of physical activity and there is evidence that physical activity levels vary by sex, race, and age subgroups [14,15]. Only 32% of youth in the United States report two or less hours of screen time per day [15]. Among adolescents, declines in physical activity have been associated with depressive symptoms [16]. A prospective cohort study indicated that sedentary behavior increases throughout adolescence and was associated with a greater risk of depressive symptoms [17]. Sedentary behavior has also been linked to anxiety symptoms among youth [18]. These studies and much of the research examining physical activity and mood in adolescents have been centered on the overall population [19,20]. Little is known about the physical activity levels and screen time of youth using specialty mental health services, including those with mood disorders, anxiety disorders, or attention-deficit/hyperactivity disorder. These youth receiving care in mental health clinics are likely to have more serious mental health concerns and belong to the group that has mental illness as adults [10]. Research indicates that adults with serious mental illness have lower levels of physical activity than the general population [21]. This study is an important step towards understanding physical activity levels in youth using specialty mental health services, which are an at-risk group for premature CVD risk. Achieving optimal cardiovascular health at the population level will require early intervention, especially among high-risk groups [4,22].

This study examines accelerometer-based physical activity levels and self-reported screen time among overweight/obese youth who were receiving specialty mental health care. The participants were enrolled in a randomized clinical trial testing a weight management program for overweight and obese youth with mental illness. The primary aim of these secondary analyses was to report baseline accelerometer-based physical activity levels and self-reported screen time among participants. A secondary aim was to examine physical activity levels and screen time among age, sex, race, and psychiatric diagnosis subgroups.

## 2. Materials and Methods

### 2.1. Study Overview

The baseline data used for these analyses were from a 12-month weight management trial for youth aged 8–18 years who were receiving specialty mental health services. The core design was a two-arm clinical trial randomized by individual, with an intervention group and a usual care control group (NCT03075306). The intervention group received a weight management program that included sessions with coaches to encourage the adoption of dietary and physical activity changes to achieve a healthy weight. These sessions were either conducted in the participant’s home or remotely using telephone and video conferencing. The primary outcome was change in BMI z-score at 12 months.

### 2.2. Participants and Settings

One hundred twelve youth aged 8–18 years with BMI z-score ≥85% for age and sex were recruited from participating outpatient mental health clinics. Participants had to be receiving specialty mental health care from a participating center for inclusion. Exclusion criteria included developmental delay precluding completion of study procedures, history of substance-related disorder or a genetic disorder of obesity, weight in excess of 400 pounds, chronic use of non-psychiatric medication associated with weight gain, ≥5% weight loss within the last 6 months, pregnancy or nursing, planning to end care at the clinic within 6 months, or plans to move from the geographic area within 12 months. These analyses included participants who met the minimum accelerometer wear time requirement as described below.

### 2.3. Measures

Parents self-reported demographics and chart review provided the primary clinical mental health diagnoses. Race was reported using the following categories: Black; Hispanic/Latino, not Black; White/Caucasian, not Hispanic/Latino; and Other. Standardized procedures were used to assess participant height and weight. Overweight was defined as an age-sex specific BMI percentile between 85% and 94% and obesity was defined as ≥95th percentile [11].

We assessed self-reported screen time using a modified screen time questionnaire [23]. This version included the original three activities (watching TV/DVDs/videos, using a computer not for homework, and using Xbox/PlayStation or other electronic games when sitting) plus an item regarding the use of cellphones or tablets. Each questionnaire item assessed h/day (0, 0.5, 1, 2, 3, 4, 5+) for an average weekday and weekend day. We calculated a weighted daily average ((5 × weekday hours + 2 × weekend hours)/7).

Physical activity was assessed using an accelerometer (Actigraph wGT3X-BT, Pensacola, FL, USA). Participants were asked to wear the accelerometer on the waist during waking hours across a seven-day period. Minimum wear time was 10 h/day for four days and we used the Evenson cut-points for physical activity intensity [24]. We reported average min/day of vigorous, moderate, and moderate-to-vigorous physical activity (MVPA).

### 2.4. Statistical Analyses

We compared between-group differences using Fisher’s exact tests for categorical variables and Welch’s *t*-test for continuous variables. We verified *t*-test results by performing sensitivity analyses for the *t*-tests, re-examining mean outcomes when extreme values (greater than 1.5 IQR) were excluded. Using separate general linear models (GLMs), an approach that allows for both categorical and continuous predictors and relaxes the normal distribution assumption, we examined the relationship between participants’ characteristics and the outcomes of MVPA and screen time while adjusting for other characteristics in the model. Due to small sample sizes in the race/ethnicity subgroups we examined the multivariable modeling focused on race. Similarly, due to sample size, the other diagnosis category was combined with the anxiety category in the multivariable modeling. The model was coded as (0, 1) for age (younger and older age groups), sex (females and males), and race (non-Black and Black), respectively. We reported results for the adjusted model. For categorical variables that had more than two categories (e.g., diagnosis), we reported a chi-square *p*-value for the overall test of association. We examined age by diagnosis interactions and only included statistically significant interaction terms in the final model. The GLM model assumptions were verified by examining the residuals of the models, and sensitivity analyses were conducted excluding extreme values. Analyses were conducted using R (R Foundation for Statistical Computing, Vienna, Austria).

### 2.5. Ethics Statement

This study was carried out in accordance with the Declaration of Helsinki and the protocol was approved by the Johns Hopkins Medicine Institutional Review Board (130511, 12/2017). Written informed consent was obtained from the parents/guardians of all participants, and additional assent was obtained from all participants.

## 3. Results

Among the 112 participants enrolled in the trial, there were 100 participants at baseline who met minimal wear-time criteria of 4 days with at least 10 h of wear time per day, forming the analysis sample of this report. Among the 100 participants in these analyses, there was an average of 5.3 days (SD = 1.1) and 13.7 h/day of wear time (SD = 1.7). All days with valid wear time were used in analyses. There were no differences in demographic characteristics between those included and those excluded from the analysis sample. The sample was 43% female, 44% Black, and 48% were aged 8–12 years (Table 1). The most prevalent primary diagnoses were attention-deficit/hyperactivity disorder (ADHD, 43%), anxiety-related disorders (28%), and depressive disorders (21%). The distributions of diagnoses were different between the age groups (*p* = 0.048), such as a higher percentage of ADHD diagnosis in the younger age group compared to the older age group. The average BMI percentile was 96.4, with 75% having a BMI percentile ≥ 95th percentile.

**Figure 1:**
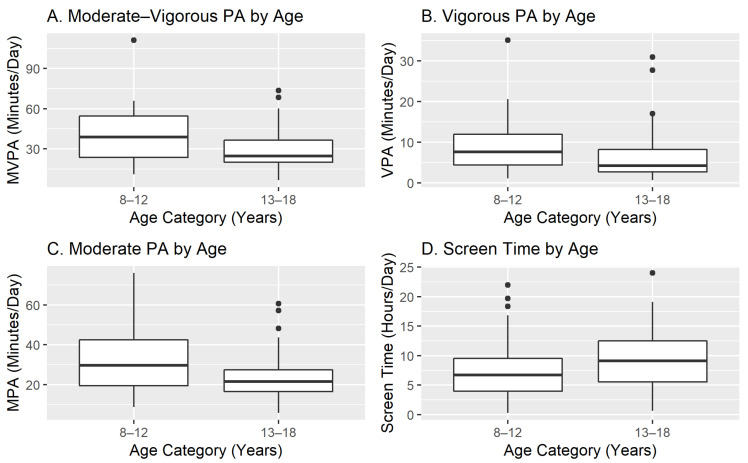
Physical activity and screen time by age group.

Figure 1 displays physical activity and screen time by age group. Overall, participants averaged 34.6 (SD = 18.1) min/day of MVPA and 8.58 (SD = 5.2) h/day of screen time. Compared to youth aged 13–18 years, the youth aged 8–12 years had higher MVPA (M = 39.9, SD = 19.2 versus M = 29.7, SD = 15.6, *p* = 0.005) and higher moderate-intensity physical activity (M = 31.2, SD = 13.9 versus M = 23.1, SD = 11.6, *p* = 0.002). The *t*-tests indicated no differences in screen time (M = 7.6, SD = 5.2 versus M = 9.5, SD = 5.1, *p* = 0.060) or vigorous physical activity (M = 8.8, SD = 6.1 versus M = 6.6, SD = 6.2, *p* = 0.080) for the younger and older age groups, respectively. Sensitivity analyses that excluded participants with extreme scores indicated no change in the *t*-test results for the physical activity but resulted in a significant difference in screen time by age group.

Table 2 shows the adjusted GLM models for the association between participant characteristics and both physical activity and screen time. Neither model had a significant diagnosis by age group interaction. Consequently, the interaction was not included in the final model. Higher levels of MVPA were associated with the younger age group (*p* = 0.012), male participants (*p* = 0.013), and having a lower BMI z-score (*p* = 0.014). Diagnosis was associated with MVPA (*p* = 0.034), and the model indicated that those with anxiety or other diagnoses had lower activity levels than those with an ADHD diagnosis. No other differences between diagnoses were identified. Higher screen time was associated with participants who were Black (*p* = 0.007). Sensitivity analyses that excluded individuals with extreme residual scores indicated no difference in the reported GLM results. Additional sensitivity analyses that included 10 participants with 3 days of ≥10 h of accelerometer wear time were also aligned with the physical activity GLM results. This sensitivity analysis resulted in higher screen time being associated with both the older age group and participants who were Black.

There were 11 participants who met the physical activity guidelines of at least 60 min/day of MVPA. Seven were in the younger age group. Eight participants met the guidelines of less than 2 h/day of screen time, six of whom were in the younger age group. The only participant who met both guidelines was in the younger age group.

## 4. Discussion

We examined physical activity and screen time at baseline in a weight management trial for overweight/obese youth aged 8–18 years with mental illness receiving specialty mental health services. Only approximately one-tenth of the study sample met physical activity guidelines of at least 60 min/day of physical activity and eight percent met the screen time recommendation of less than 2 h/day. Overall, only one percent of participants met both daily physical activity and screen time recommendations. These are lower than rates reported in the International Study of Childhood Obesity, Lifestyle, and the Environment where 7.2% of U.S. children and 14.5–20.8% of children in industrialized countries aged 9–11 years met both physical activity and screen time recommendations [15]. In the current study only 15% of 8–12-year-olds met physical activity guidelines, which is substantially lower than national estimates of between 26.5% and 42% of pre-teen youth reporting meeting physical activity guidelines [15,25]. Interestingly, only 8% of youth above the age of 12 in the general population met the guidelines, which aligned with the findings in our study [25]. This is one of the first reports of objectively measured physical activity levels among children with mental illness using specialty mental health services. These results substantiate that national concerns regarding low levels of physical activity among youth should be heightened in this group with mental illness.

Overall, 8% of participants met the recommendation of less than 2 h/day of screen time, with a higher rate reported among the younger age group (12.5%). As with physical activity, this is substantially lower than national estimates, which indicate 26.5–76.4% of children met this screen time recommendation [15,26]. Participants who were Black reported more screen time than other participants, identifying the need for tailored interventions and support. Limiting screen time is a recommended strategy for addressing obesity among youth, and evidence suggests it may also have mental health benefits [11,12,13,26,27]. As both physical activity and screen time are modifiable factors associated with obesity, they appear to be highly relevant targets for lifestyle interventions in this group.

Reports have associated depressive and anxiety-related symptoms with lower physical activity in youth, but the underlying mechanisms are not clear [17,18]. One study indicated youth with ADHD were less likely to play sports, which may contribute to lower physical activity levels [28]. A survey of teens and young adults receiving mental health services indicated interest in physical activity promotion being offered in conjunction with mental health services [29]. However, interest in physical activity was inversely related to the participant’s perception of exercise benefits and self-rated autonomy toward exercising. Parker et al. suggested the use of a collaborative problem-solving process to promote physical activity among young people using mental health services [29].

The current analyses were conducted among overweight/obese youth engaged in mental health clinics who enrolled in a weight management study. Although being in a study testing an intervention may limit the generalizability of the findings, these baseline analyses focused on youth who, because of their overweight/obesity, are likely to have a significant benefit from increased physical activity and decreased screen time. The trial was not powered for these secondary analyses, and multiple-comparison adjustments were not performed. Further research should confirm these findings with a sufficient sample size for subgroup comparisons. Longitudinal investigation may provide greater insight into the unique challenges this group faces as well as modifiable factors associated with higher physical activity and lower screen time. The authors are not aware of other reports of screen time and objectively measured physical activity among youth with mental illness in specialty mental health care. Strengths of this study included the objective assessment of physical activity and the diversity of the study population in terms of age, race, and primary diagnosis.

Based on self-report from parents, one in twenty children in the United States have problems with anxiety or depression, which has also been associated with a greater need for medical care or care coordination compared to children without anxiety- or depression-related problems [30]. Moreover, there are concerns about the long-term effect of COVID-19 on the mental health of youth [31]. Reviews consistently note that higher levels of physical activity are associated with better mental health among youth (e.g., lower anxiety symptoms, lower depressive symptoms) [32,33]. For example, youth aged 7 and 14 years who met the 60 min/day of MVPA guideline had lower risk of depression [34]. There is evidence that both regular physical activity and limited screen time have mental health benefits among youth [32,33]. Additionally, there is evidence that aerobic exercise can help reduce the symptoms of ADHD [35]. With only 11% of the participants in the current study meeting physical activity recommendations, interventions increasing daily physical activity and decreasing screen time may confer substantial mental and physical health benefits among youth using mental health services. Unfortunately, research examining the mental health benefits of physical activity for children and adolescents lags behind research among adults in this area [13,32,33]. Determining the most effective mode and dose of physical activity in order to improve the mental health and physical health of youth, particularly among those with diagnosed mental illness, deserves further investigation [13,32,33].

## 5. Conclusions

In 2015, the American Heart Association (AHA) called attention to mental illnesses in youth as important risk conditions for early CVD, and declared the need for transformational change in the screening and management of overweight and obesity in this population [4]. In addition, we need to provide early treatment of CVD risk factors in order to achieve public health objectives related to improving cardiovascular health [22]. Achieving optimal cardiovascular health at the population level not only requires early intervention, but also an understanding of high-risk groups that may need additional assistance in achieving this goal. In order to achieve equity in mental and physical health, additional efforts are needed to identify and assist youth demonstrating the greatest need. Baseline data from this study indicate that youth with overweight/obesity and mental illness who are using specialty mental health services have lower levels of physical activity and higher levels of screen time compared to published age-related estimates in the general population. These results reinforce the need for targeted lifestyle approaches for this at-risk group. Among this at-risk group, additional support may be needed for sex, age, and race subgroups.

## Figures and Tables

**Table 1:** Participant characteristics by age group.

	Total	Age in Years		
		8–12	13–18	
	N = 100	n = 48	n = 52	
	n (%)	n (%)	n (%)	*p* ^1^
**Sex**				0.317
Female	43 (43.0)	18 (37.5)	25 (48.1)	
**Income**				0.558
<$25,000	21 (21.0)	11 (22.9)	10 (19.2)	
$25,000–$49,999	18 (18.0)	10 (20.8)	8 (15.4)	
$50,000–$74,999	13 (13.0)	4 (8.3)	9 (17.3)	
≥$75,000	47 (47.0)	23 (47.9)	24 (46.2)	
**Race**				0.282
Black	44 (44.0)	21 (43.8)	23 (44.2)	
White/Caucasian	46 (46.0)	25 (52.1)	21 (40.4)	
Hispanic/Latino	5 (5.0)	1 (2.1)	4 (7.7)	
Other	5 (5.0)	1 (2.1)	4 (7.7)	
**Psychiatric Diagnosis**				0.048
ADHD	43 (43.0)	27 (56.2)	16 (30.8)	
Anxiety	28 (28.0)	11 (22.9)	17 (32.7)	
Depression	21 (21.0)	6 (12.5)	15 (28.8)	
Other	8 (8.0)	4 (8.3)	4 (7.7)	
	M (SD)	M (SD)	M (SD)	
**Body Mass Index**				
BMI z-score	2.0 (0.5)	2.0 (0.4)	2.0 (0.5)	0.600
BMI percentile	96.4 (3.6)	96.7 (3.1)	96.1 (3.9)	0.418

^1^ *p* indicates between-group differences. Fisher’s exact tests were used for categorical variables and *t*-tests were used for continuous variables.

**Table 2:** Association of physical activity and screen time with participant characteristics.

Participant Characteristic	Moderate-to-Vigorous Physical Activity			Screen Time		
	β	95% CI	*p* ^1^	β	95% CI	*p* ^1^
**Intercept**	54.2	(37.8, 70.6)	<0.001	0.6	(−4.5, 5.6)	0.829
**Age Group**						
8–12 years	ref			ref		
13–18 years	−8.5	(−15.1, −2.0)	0.012	1.9	(−0.1, 3.9)	0.063
**Sex**						
Female	ref			ref		
Male	9.4	(2.1, 16.6)	0.013	1.9	(−0.3, 4.1)	0.101
**Race**						
Non-Black	ref			ref		
Black	4.4	(−2.0, 10.9)	0.179	2.8	(0.8, 4.7)	0.007
**Diagnosis**			0.034 ^2^			0.345 ^2^
ADHD	ref			ref		
Anxiety	−9.8	(−17.7, −1.9)		1.8	(−0.6, 4.2)	
Depression	−1.6	(−11.0, 7.8)		1.0	(−1.9, 3.8)	
**BMI z-score**	−9.5	(−16.9, −2.1)	0.014	1.9	(−0.04, 4.2)	0.102

^1^ *p*-Values represent pairwise comparison with the reference category. ^2^ Overall test examining chi-square *p*-value with d.f. = 2.

## Data Availability

Deidentified data may be available to researchers whose proposed use of the data has been approved and have an appropriate data access agreement along with an IRB approval.
